# Final esthetic result of ceramic restorations cemented with different colors of cement

**DOI:** 10.1002/cre2.524

**Published:** 2022-01-09

**Authors:** Catarina Gomes, Francisco Martins, José Alexandre Reis, Carlos Pérez Albacete‐Martinez, Paulo Durão Maurício

**Affiliations:** ^1^ Health Sciences PhD Program Universidad Católica de Murcia UCAM Murcia Spain; ^2^ Interdisciplinary Research Center Egas Moniz (Ciiem) Instituto Universitário Egas Moniz (IUEM) Almada Portugal; ^3^ Oral Surgery and oral Implantology Department Universidad Católica de Murcia (UCAM) Murcia Spain

**Keywords:** ceramics, color, dental porcelain, resin cements

## Abstract

**Objective:**

The purpose of this study is to evaluate the color changes of lithium disilicate ceramics when cemented with different brands of cement by varying the thickness of the ceramic.

**Materials and Methods:**

Forty ceramic discs, shade A2, were fabricated with 0.5 and 0.8 mm thickness. Forty composite resin discs, shade A3, were also produced. The ceramic samples were cemented to the composite resin discs, with two colors of resin cement, Neutral and Warm. A spectrophotometer evaluation was made. Translucency and color change analysis was performed by calculating the Δ*E*. A two‐way analysis of variance test and multiple comparisons were performed using the Bonferroni method with a 95% confidence interval.

**Results:**

There are statistically significant differences between the two ceramic thicknesses with different brands of cement (*p* < .001). In addition, using the translucency analysis it was found that there are statistically significant differences between the two ceramic thicknesses in both types of cement (*p* < .001).

**Conclusions:**

The use of different cementation materials on lithium disilicate ceramics appears to have little visible influence at the clinical level. Different ceramic thicknesses have a clinically visible influence on the final restoration color.

## 1 INTRODUCTION

The color of natural teeth is the result of a complex phenomenon, determined by the combination of their primary and secondary optical properties. This phenomenon is influenced by several factors, such as the light source, brightness, opacity, and visual perception of the observer (Joiner, 2004).

Combining the optical properties of natural teeth with different restorative materials has become an esthetic challenge in the field of Dentistry (Turgut & Bagis, 2013). The staining of the underlying tooth, restoration core, ceramic material, and cement used may affect the final color of the ceramic restoration  (Dede et al., 2016; Sonza et al., 2021).

Nowadays, it is fundamental to deepen the understanding of the optical properties of dental materials since esthetics is increasingly the main concern for patients. Correct selection of the materials is extremely important for the clinical success of esthetic restorations (Archegas et al., 2011; Tabatabaian, 2018).

Ceramics have an optical behavior very similar to natural teeth and seek to reproduce its esthetic appearance (Li et al., 2009; Soares et al., 2005). Due to their favorable optical properties, dental ceramics have become an undeniable option, especially in the anterior region, where color and translucency play a crucial role (Dede et al., 2016).

The use of resin cement is becoming increasingly popular in clinical practice (Manso & Carvalho, 2017). Their esthetics, low solubility, and high mechanical properties make them ideal for many clinical situations (Manso & Carvalho, 2017; Yu et al., 2014).

In order to improve the esthetic result of ceramic restorations, it is important to evaluate the effect of the material thickness on its optical properties (Dozić et al., 2003; Subaşi et al., 2018). Also, the cement selection and color choice by the clinician on any ceramic restoration proves to be a critical factor in obtaining an ideal esthetic and long‐term clinical success (Chang et al., 2009; Kilinc et al., 2011; Yildirim et al., 2021).

A spectrophotometer can reveal small color differences undetectable to the human eye. This equipment measures the reflection and the transmission curve of the observed object by acquiring its spectral curve, which is limited to the color measurement in the visible spectrum range (Vichi et al., 2011). Color parameters can be quantified using a color order system, developed by the Commission on Internationale of l'Eclairage (CIELAB) in 1976. This system allows three‐dimensional color determination through three coordinates: (L*) represents the luminosity, which varies from 0 (black) to 100 (white), (a*) quantifies the color red (positive value) and green (negative value), and (b*) quantifies the color yellow (positive value) or blue (negative value) (Ahn & Lee, 2008; Dede et al., 2016; Vichi et al., 2011). The color difference (Δ*E*) can be determined by comparing the different values for each object, indicating whether the change in color is perceptible by the human eye (Bayindir & Koseoglu, 2018; Turgut & Bagis, 2011).

Lithium disilicate ceramics are becoming a choice when veneers are made (Hoorizad et al., 2021). The aim of this study is to understand the optical effect of cement and ceramic in the final esthetic result of ceramic restorations by varying the ceramic thickness and the type of cement.

## 2 MATERIALS AND METHODS

Forty composite resin disks Filtek™ Supreme XTE A3 Body Shade (3M ESPE, St. Paul, MN, USA) were obtained through a resin former (Porcelain Sampler, Ref. 7015; Smile Line, Saint‐Imier, Switzerland) with a diameter of 12 mm and a thickness of 1 mm. The samples were  light‐cured for 20 s, using the Elipar™ (3M, SaintPaul, MN, USA), at 1000 mW/cm^2^, according to the instructions of the manufacturer.

Ceramic disks (*n* = 40), with 12 mm diameter, were cut from prefabricated lithium disilicate ingots IPS e.max® Press HT shade A2 (Ivoclar Vivadent, Schaan, Liechtenstein) using an ISOMET 1000 microtome (Buehler, Lake Bluff, IL, USA) at a speed of 250 rpm, cooled with deionized water, and at a constant weight (Hoorizad et al., 2021). Ceramic samples were made with two thicknesses, 0.5 and 0.8 mm (Carrabba et al., 2020).

To ensure thickness, a digital caliper was used to check all ceramic and resin samples at three different points. All samples were polished with a LabolPol‐4 (Stuers, Cleveland, OH, USA) with sequential grinding papers (Carbimet 2; Buehler, Lake Bluff, USA) of ISO/FEPA 400, 600, and 1200 grit at a constant speed of 100 rpm (Hoorizad et al., 2021).

The ceramic samples were surface‐treated with 9.6% hydrofluoric acid (PulpDent Corporation, Watertown, MA, USA) for 90 s and rinsed with distilled water for 60 s, followed by the application of 37% orthophosphoric acid (R&S, Aubagne, France). A microbrush was used in a circular motion for 60 s before rinsing the surface with distilled water for 60 s. The samples were then cleansed for 4 min in an ultrasonic bath with distilled water (Hoorizad et al., 2021). To ensure dryness, the samples were removed from the ultrasonic bath and flushed with 96% alcohol for 30 s. The Calibra® Silane (Dentsply International, Milford, DE, USA) was applied for 20 s with a microbrush and then activated in a furnace at 100°C. Finally, the adhesive Optibond™ FL (Kerr, Scafati, Italy) was applied without photopolymerization.

The ceramic samples were randomly paired to the composite samples using the RAND() formula (Microsoft Excel, Redmond, WA, USA) and divided into 4 groups with 10 samples each.

For each thickness, one group was cemented with either Variolink® Esthetic LC (Ivoclar Vivadent, Schaan, Liechtenstein) color Neutral or Warm.

The cemented samples were placed between two glass plates and a constant pressure of 20 N was made using a weight of 2 kg, for 60 s, in order to standardize the cement thickness (Carrabba et al., 2020; Hoorizad et al., 2021; Tabatabaian et al., 2018; Tomaselli et al., 2019). A light cure was performed through the glass plate for 60 s using the same light source, as described previously.

After this procedure, all samples were placed in a dry environment at room temperature and in the absence of light for 24 h.

The color was determined according to the CIELAB color scale relative to the standard illuminant D65 on a reflection Spectro Shade spectrophotometer (Spectro Shade; MHT S.p.A., Milan, Italy) for each ceramic sample before and after its cementation on a gray, black, and white background (Bayindir & Koseoglu, 2018; Carrabba et al., 2020).

Color difference (Δ*E*) was determined by the values of *L, a*, and *b*, obtained by the spectrophotometer on the readings of the samples on the gray background before and after cementation. The color difference (Δ*E*) was calculated through the following formula (Bayindir & Koseoglu, 2018):

$$∆E=\sqrt{{\left(∆L^{*}\right)}^{2}+{\left(∆a^{*}\right)}^{2}+{\left(∆b^{*}\right)}^{2}}.$$

Translucency parameter (TP) was calculated for the cemented samples by the values of *L, a*, and *b*, obtained through the spectrophotometer against the white and black backgrounds for the same sample, by the following formula (Bayindir & Koseoglu, 2018):

$$\mathrm{TP}=\sqrt{{\left(L_{w}-L_{b}\right)}^{2}+{\left(a_{w}-a_{b}\right)}^{2}+{\left(b_{w}-b_{b}\right)}^{2}},$$

where *L*_b_, *a*_b_, and *b*_b_ represent the readings on the black background and *L*_w_, a_w_, and *b*_w_ represents the readings on the white background.

The statistical analysis was carried out through a database designed in the program Statistics Package for the Social Sciences, version 20.0 (IBM, Inc., Chicago, IL, USA). A two‐way analysis of variance test and multiple comparisons were performed using the Bonferroni method with a 95% confidence interval.

Being an in vitro study, the present study does not violate the ethical rights of animals or humans.

## 3 RESULTS

The results concerning the influence of cement color and substrate on the final color of the restoration (Table 1) indicate that the group with the highest value of Δ*E* is the thinnest ceramic, cemented with Variolink® color Neutral (16.12). On the other hand, the lowest value presented, 10.50, is represented by the thickest ceramic, cemented with Variolink® color Neutral.

**Table 1:** Calculation of the mean value of Δ*E* and standard deviation between samples of cemented ceramics and the initial ceramic samples

	Thicknesses of ceramics		
Cement	0.5 mm	0.8 mm	
Variolink® Neutral	16.12 ± 1.05	10.50 ± 1.42	*p* < .001^a^, *
Variolink® Warm	15.59 ± 1.12	10.86 ± 0.67	*p* < .001^a^, *
	*p* = .286^a^	*p* = .468^a^	

^a^ Two‐way ANOVA and multiple comparisons (Bonferroni).

* Statistically significant difference for a 95% confidence interval.

Regarding the influence of cement color and ceramic thickness on the final color of the restoration (Table 2), the group with the highest mean value of Δ*E* is the thickest ceramic, cemented with the Variolink® color Neutral (4.00) and the lowest mean value is 0.058 in the thinnest ceramic, cemented with Variolink® color Warm.

**Table 2:** Calculation of the mean value of Δ*E* and standard deviation between cemented ceramic samples and composite resin samples

	Thicknesses of ceramics		
Cement	0.5 mm	0.8 mm	
Variolink® Neutral	0.11 ± 0.19	4.00 ± 1.09	*p* < .001^a^, *
Variolink® Warm	0.058 ± 1.12	3.90 ± 0.57	*p* < .001^a^, *
	*p* = .691^a^	*p* = .726^a^	

^a^ Two‐way analysis of variance and multiple comparisons (Bonferroni).

* Statistically significant difference for a 95% confidence interval.

The results concerning the influence of cement color and ceramic thickness in the translucency of the final restoration (Table 3) indicate that the group with the highest value of TP is the thinnest ceramic, which is cemented with Variolink® color Neutral (16.77). On the other hand, the lowest value presented, 12.07, occurs in the thickest ceramic, which is cemented with Variolink® color Neutral.

**Table 3:** Calculation of the mean value of Δ*E* and standard deviation of the TP

	Thicknesses of ceramics		
Cement	0.5 mm	0.8 mm	
Variolink® Neutral	16.77 ± 0.63	12.07 ± 1.08	*p* < .001^a^, *
Variolink® Warm	15.86 ± 1.86	12.53 ± 0.80	*p* < .001^a^, *
	*p* = .093^a^	*p* = .393^a^	

Abbreviation: TP, translucency parameter.

^a^ Two‐way analysis of variance and multiple comparisons (Bonferroni).

* Statistically significant difference for a 95% confidence interval.

The results indicate that there is no statistically significant interaction between the study variables at a value of *p*= .207 (Table 1), *p*= .974 (Table 2) and *p*= .075 (Table 3). Nonetheless, by analyzing the results obtained it is possible to verify that by comparing thicknesses, the thinnest ceramic has the highest mean value of Δ*E* with either cement (Tables 1 and 3) and Variolink® color Neutral has the highest average results (Tables 1, 2, 3). On the other hand, on the thickest ceramic, the cement with the highest average results is Variolink® color Warm (Tables 1 and 3).

Considering both types of cement, there are statistically significant differences (*p* < .001) between the two ceramic thicknesses (Tables 1, 2, 3). Although there are no statistically significant differences (*p* > .05) between the two types of cement for either ceramic thickness (Tables 1, 2, 3).

## 4 DISCUSSION

Lithium disilicate ceramics were chosen not only for their esthetics but also for their mechanical and optical properties. Their study has a clinical added value when associated with different types of resin cement (Conrad et al., 2007; Ho and Matinlinna, 2011; Hoorizad et al., 2021). The substrate used for cementation of the ceramic consisted of 1‐mm‐thick composite resin discs, similar to previous studies in order to standardize color (Chen et al., 2012; Lehmann et al., 2017).

A spectrophotometer has been considered by several authors to be the method with the greatest accuracy and clinical applicability available (Chen et al., 2012; Lehmann et al., 2017). As with previous studies, the data obtained by the spectrophotometer through the CIELab system and analyzed through the calculation of Δ*E* allow the calculation of the differences in color and translucency between the various samples (Archegas et al., 2011; Kürklü et al., 2013; Turgut & Bagis, 2013). There is no consensus in color changes perceptible by the clinician and the values of Δ*E* (Chang et al., 2009; Chen et al., 2015; Da Silva et al., 2008; Vichi et al., 2011). Da Silva et al. (2008) considered in their study that the color difference is clinically noticeable when Δ*E* is >2.69, whereas Chang et al. (2009) reported a value of Δ*E* = 2.0. Vichi et al. (2011) published the lowest perceptibility value, a Δ*E* of 1.0 and Hoorizad et al. (2021) considered Δ*E* ≤ 3.3 as clinically acceptable. In the present study, it was considered that the color difference is clinically perceptible when Δ*E* > 1.7, as in the study of Douglas et al. (2007).

Concerning the color difference (Δ*E*), it is possible to verify that for both thicknesses, there are no clinically detectable differences between the tested cement on the final color of the restoration (Tables 1 and 2).

It was found that the color variation of the cement has no influence on the final color of the restoration (Δ*E* < 1.7). These results agree with the studies of Turgut and Bagis (2013) and Carrabba et al. (2020).

On the other hand, it is possible to verify that using cement Variolink® Warm or cement Variolink® Neutral, there are clinically detectable differences when there is a variation in the ceramic thickness (Tables 1 and 2). These results suggest that a 0.3 mm variation of the ceramic thickness has an influence on the final color of the restoration (Δ*E* > 1.7) and is in agreement with Azer et al. (2011), Xing et al. (2017), and Tomaselli et al. (2019).

For the TP the results for both thicknesses indicate no clinically detectable differences (TP < 1.7), as observed by the observer in the final color of the restoration between the use of the studied cement, and as concluded in the studies of Dozić et al. (2003), Douglas et al. (2007), Chaiyabutr et al. (2011), Xing et al. (2017), and Czigola et al. (2019).

However, it is possible to verify that there are clinically noticeable differences (TP > 1.7), when there is a variation of the ceramic thickness, which suggests that there is an increase of the translucency with the decrease of the ceramic thickness as demonstrated by Turgut and Bagis (2013), Kürklü et al. (2013), and Chen et al. (2015).

The optical properties of the ceramics are influenced by the thickness of the ceramic and are not influenced by the color of the cementing material. Further studies will be required to evaluate the optical behavior of materials when cemented with different substrate colors and different brands of cement.

## 5 CONCLUSIONS

Within the limitations of this in vitro study, the following conclusions were obtained:

1. Different lithium disilicate ceramic thicknesses have a clinically visible influence on the final restoration color.
2. The use of different cementation materials on lithium disilicate ceramics appears to have little visible influence at the clinical level.

## CONFLICT OF INTERESTS

The authors declare that there are no conflict of interests.

## AUTHOR CONTRIBUTIONS

All authors contributed to the conception and design of this study. Material preparation, data acquisition, and preparation of the manuscript were performed by Catarina Gomes. Research design, data analysis, and interpretation were performed by Francisco Martins and José Alexandre Reis. Interpretation and manuscript revision were performed by Carlos Pérez Albacete‐Martinez and Paulo Durão Maurício. All the authors read and approved the final manuscript.

## Data Availability

The data that support the findings of this study are available from the corresponding author upon reasonable request.
